# Chimeric Proteins Containing MAP-1 and Functional Domains of C4b-Binding Protein Reveal Strong Complement Inhibitory Capacities

**DOI:** 10.3389/fimmu.2018.01945

**Published:** 2018-08-28

**Authors:** Cecilie E. Hertz, Rafael Bayarri-Olmos, Nikolaj Kirketerp-Møller, Sander van Putten, Katrine Pilely, Mikkel-Ole Skjoedt, Peter Garred

**Affiliations:** ^1^Laboratory of Molecular Medicine, Department of Clinical Immunology Section, Rigshospitalet, Faculty of Health and Medical Sciences, University of Copenhagen, Copenhagen, Denmark; ^2^Finsen Laboratory, Rigshospitalet, Biotech Research and Innovation Center (BRIC), Faculty of Health and Medical Sciences, University of Copenhagen, Copenhagen, Denmark

**Keywords:** complement activation, lectin pathway, classical pathway, MAP-1, C4BP, complement inhibition, chimeric protein

## Abstract

The complement system is a tightly regulated network of proteins involved in defense against pathogens, inflammatory processes, and coordination of the innate and adaptive immune responses. Dysregulation of the complement cascade is associated with many inflammatory disorders. Thus, inhibition of the complement system has emerged as an option for treatment of a range of different inflammatory diseases. MAP-1 is a pattern recognition molecule (PRM)-associated inhibitor of the lectin pathway of the complement system, whereas C4b-binding protein (C4BP) regulates both the classical and lectin pathways. In this study we generated chimeric proteins consisting of MAP-1 and the first five domains of human C4BP (C4BP^1−5^) in order to develop a targeted inhibitor acting at different levels of the complement cascade. Two different constructs were designed and expressed in CHO cells where MAP-1 was fused with C4BP^1−5^ in either the C- or N-terminus. The functionality of the chimeric proteins was assessed using different *in vitro* complement activation assays. Both chimeric proteins displayed the characteristic Ca^2+^-dependent dimerization and binding to PRMs of native MAP-1, as well as the co-factor activity of native C4BP. In ELISA-based complement activation assays they could effectively inhibit the lectin and classical pathways. Notably, MAP-1:C4BP^1−5^ was five times more effective than rMAP-1 and rC4BP^1−5^ applied at the same time, emphasizing the advantage of a single inhibitor containing both functional domains. The MAP-1/C4BP chimeras exert unique complement inhibitory properties and represent a novel therapeutic approach targeting both upstream and central complement activation.

## Introduction

The complement system constitutes a central effector arm of the vertebrate immune system occupying a pivotal position as an early danger sensor and mediator of immunological and inflammatory processes (1, 2). Complement is activated via three distinct pathways, i.e., the classical (CP) the lectin (LP) and the alternative pathway (AP) that converge on the generation and deposition of opsonins, release of anaphylatoxins, and generation of the terminal complement complex (TCC) which all together help coordinating the cellular and humoral immune responses (3–6).

Initiation of CP is mediated mainly by the binding of C1q to immune-complexes, but also to conserved pathogen-specific structures, altered self-antigens or interaction with the pentraxins (7) and leads to the activation of the serine proteases C1r and C1s (8, 9). The LP is initiated by two groups of pattern recognition molecules (PRM): c-type lectins, such as mannose-binding lectin (MBL), collectin-10 (CL-10, CL-L1), and collectin-11 (CL-11, CL-K1) and ficolins, i.e., ficolin-1 (M-ficolin), ficolin-2 (L-ficolin), and ficolin-3 (H-ficolin or Hakata antigen). Binding of PRMs to ligands activates the MBL-associated serine proteases (MASPs), causing cleavage and activation of C2 and C4 (10). The AP is constantly “probing” surfaces in a non-specific fashion by means of a constitutive low level of spontaneous hydrolysis of C3 into the C3b analog C3(H_2_O) (11). Maybe more importantly, C3b generated from any of the three pathways is amplified via the AP. In fact, AP amplification may account for up to 90% of the total complement activation independently of the triggering pathway (7, 12). Whether the AP can be activated via a similar PRM-dependent mechanism involving properdin remains controversial (13, 14).

Complement is much more than a pathogen-sensing system, with ties spanning into coagulation, inflammation and embryonic development (2). In order to carry out its diverse functions it relies on a delicate balance between activation and inhibition by means of a set of membrane-bound and soluble regulatory proteins (15), the latter including the LP inhibitor MBL/ficolin/CL-associated protein 1 (MAP-1, also named MAp44), and the LP and CP regulators C4b-binding protein (C4BP) and C1 inhibitor (a protease inhibitor of the serpin super family that inactivates C1r, C1s, MASP-1, and MASP-2) (16).

Two genes encode all five naturally-occurring MASP isoforms in mammals (17): three proenzymes (i.e., MASP-1, -2, and -3) and the two non-proteolytic proteins sMAP (MAp19) and MAP-1. The proteolytic MASPs consist of a heavy chain—comprised of two CUB domains separated by an epidermal growth factor (EGF) domain, and two complement control protein/short consensus repeats (CCP/SCR) modules—and a light chain composed of the serine protease domain. MAP-1 differs from the proteolytic MASPs in that it lacks the second SCR and SP domain and thus it cannot activate complement. MAP-1 competes with the MASPs in binding to recognition molecules of the LP leading to decreased complement activation. This has been demonstrated *in vitro* (18–20), and in multiple *in vivo* disease models (21, 22).

C4BP is a soluble protein encoded in the regulator of complement activation (RCA) gene locus of chromosome 1 (23) and possesses a unique structure among the RCA proteins in being a polymer composed of several CCP containing polypeptides. The most abundant isoform in the circulation is composed of seven identical α-chains (75 kDa each) and one β-chain (45 kDa) linked together by a central core and found in a high affinity complex with the anticoagulant vitamin K-dependent protein S (24, 25). The complement regulatory functions of C4BP are located within the first CCP domains of the α-chains. C4BP binds to the negatively-charged surface of C4b via the first three CCP domains of the α-chain preventing the assembly of the classical and lectin pathways C3 convertases (26, 27). Additionally, C4BP acts as a cofactor in the complement factor I (fI)-mediated proteolytic inactivation of both soluble and membrane bound C4b (28–30). By binding to C3b via the first 4 CCP domains of the α-chain, C4BP also participates in the fI-dependent C3b degradation to iC3b in the fluid phase (31). Although it is difficult to speculate upon the genuine physiological role of the inhibitory function of C4BP since no C4BP deficiency has been diagnosed in humans (32), C4BP injected peritoneally has been shown to alleviate inflammation and tissue damage in collagen- and collagen antibody-induced arthritis mouse models (33).

Since the US Food and Drug Administration approval of the first complement-specific drug in 2007 (34), rational modulation of the complement cascade using complement inhibitors has gradually demonstrated its potential as a drug discovery strategy and therapeutic treatment (35). Especially recombinant chimeric proteins targeting different levels of the cascade are of great interest in complement-mediated therapy and have previously been created with success (36, 37). Here we aimed to create a dual inhibitor with the ability to target initial activation by both the lectin and classical pathways by combining full length MAP-1 with the first five N-terminal CCP domains of the α-chain of C4BP. This could provide a unique platform for a novel class of complement inhibitor and thus contribute to the emerging field of complement therapeutics.

## Materials and methods

### Buffers

The following buffers were used: PBS (0.2 M Na_2_HPO_4_, 35 mM K_2_HPO_4_, 0.15 M NaCl, 15 mM KCl), PBS/NaCl (0.2 M Na_2_HPO_4_, 35 mM K_2_HPO_4_, 0.5 M NaCl, 15 mM KCl), TBS/Ca^2+^ and TBS/Tw/Ca^2+^ (20 mM Tris-HCl, 150 mM NaCl, 5 mM CaCl_2_, with/without 0.05% Tween-20), TBS/EDTA and TBS/Tw/EDTA (20 mM Tris-HCl, 150 mM NaCl, 10 mM EDTA, with/without 0.05% Tween), VBS/Tw and sample buffer (4 mM C_8_H_11_N_2_NaO_3_, 145 mM NaCl, 2.6 mM CaCl_2_, 2 mM MgCl_2_, with 0.05% Tween-20 or 0.5% BSA respectively).

### Design of chimeric proteins and transfection

The coding sequences for MAP-1 (NM_001031849.2) and C4BP (NM_000715.3) were optimized for expression in Chinese hamster ovarian (CHO) cells in terms of codon adaptation index, mRNA stability, GC content, removal of cryptic splice sites, and repeats, 5' UTR, and signal peptide. All DNA manipulations were performed in Visual Gene Developer (38). MAP-1:C4BP^1−5^ comprises the coding sequence of MAP-1 followed by the first five CCP domains of the α-chain of C4BP (C4BP^1−5^). The reverse construct C4BP^1−5^:MAP-1 was designed with the coding sequence of C4BP^1−5^ located in the N-terminus of MAP-1 and separated by a flexible glycine serine linker (G_4_S)_3_. A control construct consisting of C4BP^1−5^ alone was designed with a C-terminal hexa histidine-tag. In all cases, the signal peptide of MAP-1 was used to ensure secretion. The chimeric constructs were cloned into the pcDNA5/FRT vector and transfected into Flp-in CHO cells (both from Invitrogen, Thermo Fisher Scientific, USA) according to the manufacturer's instructions. Positive transfectants were selected for Hygromycin resistance in complete medium with Ham's F-12 Nutrient Mix, 10% fetal calf serum, 2 mM L-Glutamine, 1% Penicillin-Streptomycin (Sigma-Aldrich, USA), and 500 mM of Hygromycin B (all from Gibco, Thermo Fisher Scientific unless otherwise stated), and maintained at 37°C in a humidified atmosphere with 5% CO_2_.

### Purification of recombinant proteins

The chimeric constructs were purified by immunoaffinity chromatography using the monoclonal antibody (mAb) anti-MAP-1 20C4 (39) coupled to cyanogen bromide-activated-sepharose 4B (Sigma-Aldrich). Briefly, filtered cell culture supernatants were applied to the column and washed in PBS/NaCl. Bound protein was eluted with 0.5% (w/v) citric acid (pH 2.5) and neutralized with 1 M Tris-HCl (pH 9). Elution fractions were applied on a NuPAGE bis-tris 4–12% polyacrylamide gel (Invitrogen), followed by a direct protein stain with Instant Blue (Expedeon LTD, UK) to assess the purity. The concentration of the chimeric proteins was interpolated by ELISA using rMAP-1 as standard. rC4BP^1−5^ was purified with a HisTrap excel column (GE Healthcare, Denmark) according to the manufacturer's instructions.

### SDS-page and western immunoblotting

SDS-PAGE was performed using NuPAGE bis-tris 4–12% gels with or without reducing agent and blotted onto Hybond^TM^-ECL nitrocellulose membranes (GE Healthcare) according to the manufacter's instructions. The membranes were probed with 0.5 μg/ml anti-MAP-1 20C4 or 0.3 μg/ml anti-human C4BP polyclonal antibody (pAb; Abnova, 722-D01P, Taiwan) overnight, followed by 1 h incubation with 0.4 μg/ml rabbit anti-mouse-HRP pAb (Dako, P0260, Denmark) or a 1:4000 dilution of donkey anti-rabbit-HRP pAb (GE Healthcare, NA934V) for MAP-1 and C4BP, respectively. The membranes were developed with SuperSignal WestFemto (Thermo Fisher Scientific) and analyzed with Microchemi (Bio-imaging systems, Israel).

### Fluid phase C4BP cofactor assay

The cofactor activity of C4BP, MAP-1:C4BP^1−5^, and C4BP^1−5^:MAP-1 was assessed with an established fluid phase cofactor activity assay (40, 41) measuring the fI-cofactor activity of C4BP in the proteolytic inactivation of C4b and C3b. Briefly, C4b and C3b were incubated with fI, complement factor H (fH), and equimolar concentrations of full length C4BP (all from Complement Technology Inc., USA), recombinant MAP-1 (rMAP-1, produced in house), MAP-1:C4BP^1−5^, or C4BP^1−5^:MAP-1 for 2 h at 37°C. The reactions were stopped with LDS sample buffer and reducing agent (both from Invitrogen). Samples were subjected to electrophoresis and immunoblotting as described above. Cleavage of C3b was visualized by incubation with 1.6 μg/ml rabbit anti-human C3d pAb (Dako, A006302) followed by donkey anti rabbit-HRP (GE Healthcare, NA934V) in a 1:5000 dilution. C4b cleavage was assessed by incubation with 0.3 μg/ml mouse anti-human C4d mAb (Thermo Fischer Scientific) and 0.4 μg/ml rabbit anti mouse-HRP (Dako). Membranes were developed as described previously.

### Size exclusion chromatography (SEC)

The chimeric proteins (50 μg) were applied to a Superdex 200 HR 10/30 column (GE Healthcare) with a flow rate of 0.5 ml/min in TBS/Ca^2+^ or TBS/EDTA. The eluate was collected in 1 ml fractions and analyzed by ELISA (see below).

### ELISA

#### Assessment of Ca^2+^-dependent dimerization by sandwich ELISA following SEC

Microtiter wells (MaxiSorp plate; Thermo Fisher Scientific) were coated with anti-C4BP pAb (2 μg/ml) in PBS overnight at 4°C. SEC fractions were applied in a two-fold dilution starting at 1:20 and incubated for 2 h. Biotinylated 8B3 mAb (2 μg/ml), recognizing the common heavy chain of MASP-1, MASP-3 and MAP-1 (39), was used as detection antibody for 2 h. Streptavidin-HRP conjugate (Sigma Aldrich, RPN1231V) (1:2000) was added for 1 h and the plates were developed with substrate solution TMB One (Kem Em Tek, Denmark). The reaction was stopped with 0.2 M H_2_SO_4_ and the absorbance was measured at 450 nm using an ELx80 absorbance reader (BioTek, USA).

#### Binding of chimeric proteins to ligand-bound MBL and CL-11

Microtiter wells were coated with 10 μg/ml mannan (Sigma Aldrich) in PBS overnight at 4°C followed by incubation with recombinant MBL or recombinant CL-11 (rMBL and rCL-11 produced in house; 2 μg/ml) (42). Serial dilutions of MAP-1:C4BP^1−5^, C4BP^1−5^:MAP-1, rMAP-1, and rC4BP^1−5^ in TBS/Tween/Ca^2+^ or TBS/Tween/EDTA starting at 13.5 nM were allowed to complex with rMBL for 2 h. Detection and development were done as described above. Binding of rMBL and rCL-11 to mannan was confirmed using biotinylated anti-MBL Hyb-131-1 mAb and anti-CL-11 Hyb-15 mAb (2 μg/ml), respectively.

#### Complement activation assays

Inhibition of complement activation via the LP was determined by an established ELISA-based assay (42) using specific antibodies to detect the activated and deposited products of C4, C3, and TCC. The chimeric proteins ability to inhibit complement was tested in MBL defect serum (43) and normal human serum (NHS). Mannan (10 μg/ml) was immobilized onto microtiter plates and incubated in PBS overnight at 4°C followed by incubation with 2 μg/ml rMBL for 1.5 h. Serial dilutions of rMAP-1, C4BP^1−5^:MAP-1, and MAP-1:C4BP^1−5^ starting at 667 nM were incubated with mannan/rMBL complexes for 1 h prior to addition of 2% MBL defect serum. When using NHS as a complement source, serial dilutions of C4BP^1−5^:MAP-1, MAP-1:C4BP^1−5^, MAP-1, and purified C4BP, starting at 667 nM, were added to round bottom non-absorbent titration plates (Thermo Fisher Scientific) with 2% NHS. The serum-protein mix was incubated 30 min at 4°C, transferred to mannan (10 μg/ml) coated plates and incubated for 45 min at 37°C. Detection of deposition fragments was quantified with 2 μg/ml biotinylated mouse mAbs anti-human C4 (Anti-C4) (Bioporto Diagnostics, Denmark, Hyb 162-02), 2 μg/ml anti-human C3bc BH6 (44), and 1 μg/ml anti-human C5b-9 (anti-TCC; Bioporto, Dia 011–01) incubated for 2 h. Finally, Streptavidin-HRP conjugate was added in a 1:2000 dilution for 1 h. Unless otherwise specified, all washes and incubation steps were done with VBS/Tw. Development was performed with substrate solution TMB One. Data are represented as inhibition (%), calculated as [(OD_inhibitor_ – OD_background_)/(OD_noinhibitor_ – OD_background_)]100.

#### Total complement screen

The Wieslab^TM^ Complement System Screenkit COMPL 300 (Euro diagnostica, Sweden) (45), a standardized immunoassay for measuring TCC formation by all three complement pathways, was used to assess the inhibitory capabilities of the chimeric proteins beyond the LP. MAP-1:C4BP^1−5^, C4BP^1−5^:MAP-1, rMAP-1, rC4BP^1−5^, and rMAP-1 plus rC4BP^1−5^ (starting at 667 nM for LP and CP, and at 2667 nM for AP) were applied in a two-fold dilution in non-absorbent titration plates in the respective buffers for each pathway provided with the kit. NHS in a 1:100 dilution for the CP and LP and a 1:20 for the AP was co-incubated with the inhibitors for 30 min at 4°C. Protein/serum mixes were transferred to pre-coated 96-well microtiter plates and incubated 1 h at 37°C. Following incubation the samples were analyzed with the Wieslab Complement System Screenkit according to the instruction from the manufacturer. Data are represented as described in the previous paragraph without background subtraction.

### Flow cytometry

Complement activation on kidney cells was studied by flow cytometry using HK-2 cells (ATCC, CRL-2190), an immortalized human proximal tubular epithelial cell (PTEC) line reported to be a suitable alternative to freshly isolated PTCs (46). Cells were cultured in Keratinocyte Serum Free Medium supplemented with bovine pituitary extract, human recombinant epidermal growth factor (all from Gibco) and 0.5% Penicillin-Streptomycin (Sigma-Aldrich). On the day of the experiments, the cells were detached with TryPLE Express Enzyme, washed with pre-warmed Hank's balanced salt solution (both from Gibco) with calcium and magnesium, and transferred to polystyrene round bottom tubes (Corning, USA). Data was collected using a Gallios flow cytometer and analyzed by the Kaluza software 1.2 (both from Beckman Coulter, USA). Values are reported as median fluorescence intensities (MFI). To ensure analysis of single cells, a forward scatter area vs. height gate was defined upon a uniform gated population on the forward vs. side scatter (see Supplementary Figure 1). A total of 10,000 events were recorded per experimental condition.

#### Binding of MBL to kidney cells

HK-2 cells (1.0 × 10^5^ cells/test) were incubated with serial dilutions of rMBL for 30 min at 4°C in sample buffer with or without EDTA. Prior to the addition of the detection antibody, the cells were fixed in a 1% solution of paraformaldehyde (Sigma Aldrich) for 10 min. Bound rMBL was detected using anti-human MBL Hyb 131-11 mAb (2 μg/ml) for 30 min plus goat anti-mouse PE conjugate (Sigma, P9670) in a 1:20 dilution for 20 min in the dark. The cells were washed with sample buffer at 300 × g for 5 min in between steps and all manipulations were performed at 4°C.

#### Complement deposition on kidney cells

HK-2 cells (1.0 × 10^5^ cells/test) were incubated with rMBL for 30 min, followed by 10% MBL defect serum for 1 h. The cells were fixed with 1% paraformaldehyde for 10 min. Surface-bound C4 was measured with biotinylated anti-human C4c pAb (2 μg/ml) (Dako, Q036905) for 30 min followed by Streptavidin APC conjugate (1:400) (Invitrogen, SA1005) for 20 min in the dark. To study the impact of the chimeric inhibitors on C4 deposition, serial dilutions of MAP-1:C4BP^1−5^, and C4BP^1−5^:MAP-1, rMAP-1, and C4BP^1−5^ (200 nM) were allowed to react with 10% MBL defect serum for 20 min prior to incubating with the rMBL-bound HK-2 cells for 1 h. After fixing the cells C4 deposition was measured as described previously. All washes and incubation steps were done in sample buffer at 4°C.

### Statistics

All statistical analyses were performed in GraphPad Prism software 7.02 (GraphPad, USA). Kd values (i.e., ligand concentration required to achieve half of the maximum binding) were calculated using the equation specific binding with Hill slope after subtracting the background OD and constraining the Bmax (maximum number of binding sites) to a value shared between all inhibitors (**Figure 3**). The half maximal inhibitory concentration (IC50) was calculated using a global nonlinear regression with the equation inhibitor concentration vs. response constraining the top and bottom parameters to equal 100 and 0 respectively (**Figures 4**, **5**, **8**). The significance of inhibitory differences was analyzed using one-way ANOVA with Tukey's corrections for multiple comparisons on the best-fit IC50 values from data sets with an acceptable goodness-of-fit (adjusted R-squared > 0.8). Significance of the binding of rMBL and MBL-dependent C4 deposition on the surface of HK-2 cells was assessed using a two-tailed *t*-test, while inhibition was analyzed using multiple unpaired *t*-tests with the Holm-Sidak correction for multiple comparisons. Data are represented as mean ± SEM of three independent experiments.

## Results

### Production and purification of chimeric proteins

**Figure 1:**
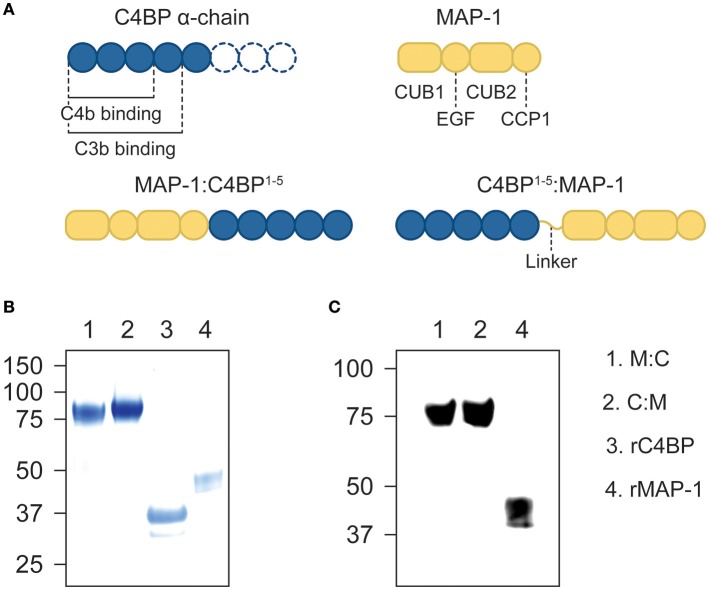
Recombinant constructs and purified proteins. **(A)** Diagram of the domain distribution of MAP-1, C4BP α-chain and the recombinant proteins. **(B)** Instant blue stain of purified MAP-1:C4BP^1−5^, C4BP^1−5^:MAP-1, and C4BP^1−5^via affinity chromatography. **(C)** The identity of the protein bands was confirmed by Western blotting using anti-MAP-1 mAb and rMAP-1 as positive control.

We designed two differently-oriented chimeric inhibitors: MAP-1:C4BP^1−5^, with the first five CCP domains of the α-chain of C4BP (alias C4BP^1−5^) located in the C-terminus of MAP-1; and C4BP^1−5^:MAP-1, where C4BP^1−5^ was placed in the N-terminus of MAP-1 via a flexible glycine-serine linker. A control protein, C4BP^1−5^ without MAP-1, was designed with a terminal hexa His-tag (Figure 1A). All three constructs were cloned into a pcDNA5/FRT plasmid and transfected into Flp-In CHO cells. MAP-1-containing chimeric proteins were purified by antibody affinity chromatography and C4BP1-5 was purified via immobilized metal ion affinity chromatography on a HisTrap excel column. Under reducing conditions, the chimeric proteins migrated as a single band with an apparent molecular weight of ~80 kDa, equivalent to the sum of rMAP-1 and rC4BP^1−5^ molecular weights (i.e., ~44 kDa and ~37 kDa respectively; Figure 1B). The identity of the protein bands was confirmed by Western blotting (Figure 1C).

### MAP-1:C4BP^1−5^ and C4BP^1−5^:MAP-1 form dimers and associate with the collectins of the LP in the presence of calcium

**Figure 2:**
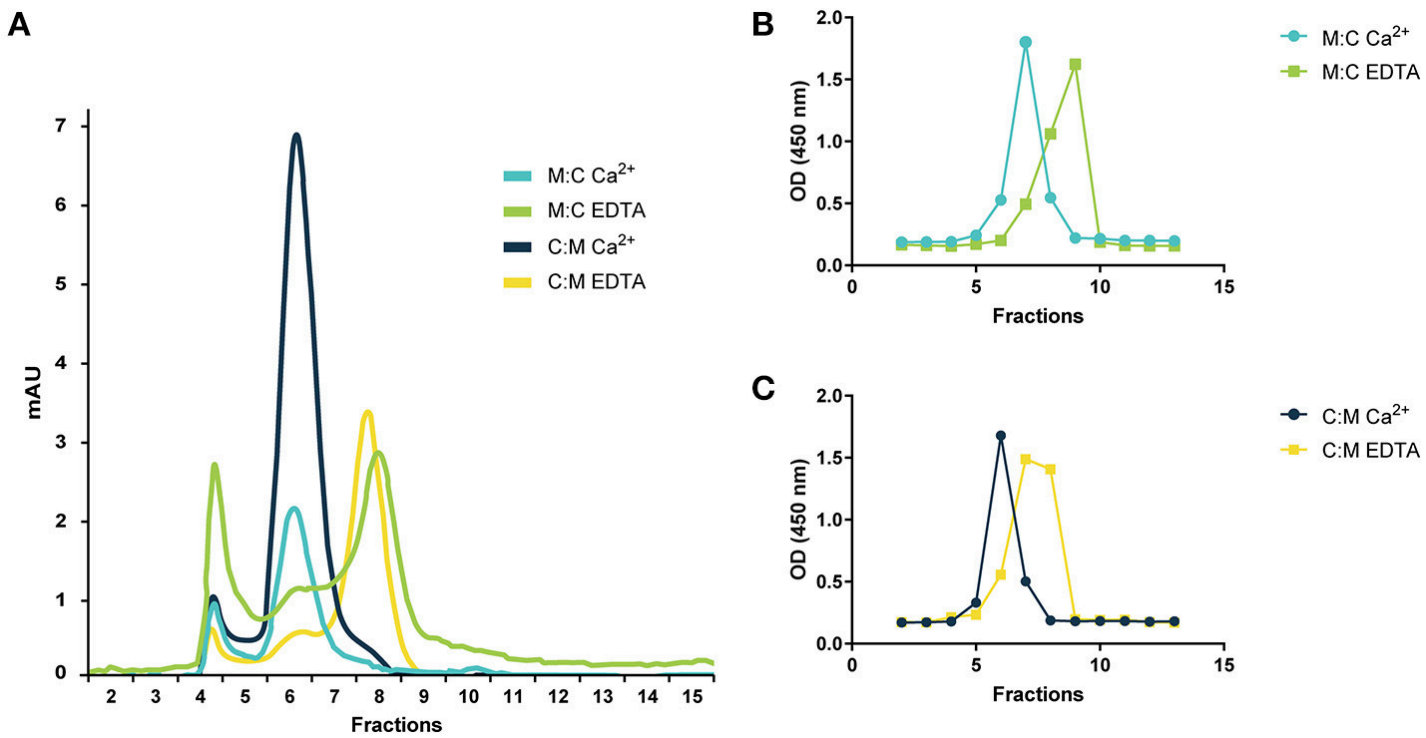
Gel filtration chromatography of chimeric proteins. **(A)** Gel filtration profile of MAP-1:C4BP^1−5^ (M:C) and C4BP^1−5^:MAP-1 (C:M) under physiological calcium conditions or 10 mM EDTA. The identity of the gel filtration profiles was confirmed by analyzing the elution fractions of M:C **(B)** or C:M **(C)** by sandwich ELISA using anti-C4BP as capture antibody and anti-MASP-1/-3/MAP-1 as detection. Relative abundance of the chimeric proteins in the elution fractions following gel filtration under calcium conditions or EDTA is expressed as OD. mAU, milli absorption units.

The MASPs are known to form head-to-tail dimers via the interaction of the CUB1 and EGF domains and to associate with the recognition molecules of the LP in a Ca^2+^-dependent manner (18, 47, 48). To study the effect of calcium in the dimerization of our recombinant inhibitors, we performed gel filtration with and without EDTA, a Ca^2+^-chelating agent. Both chimeric proteins eluted in a single overlapping peak in fractions 6–8 under physiological calcium concentrations. In the presence of EDTA, the previous peak was drastically reduced and a new one appeared in fractions 7–9 indicating that both are capable of assembling into dimers stabilized by Ca^2+^ (Figure 2).

**Figure 3:**
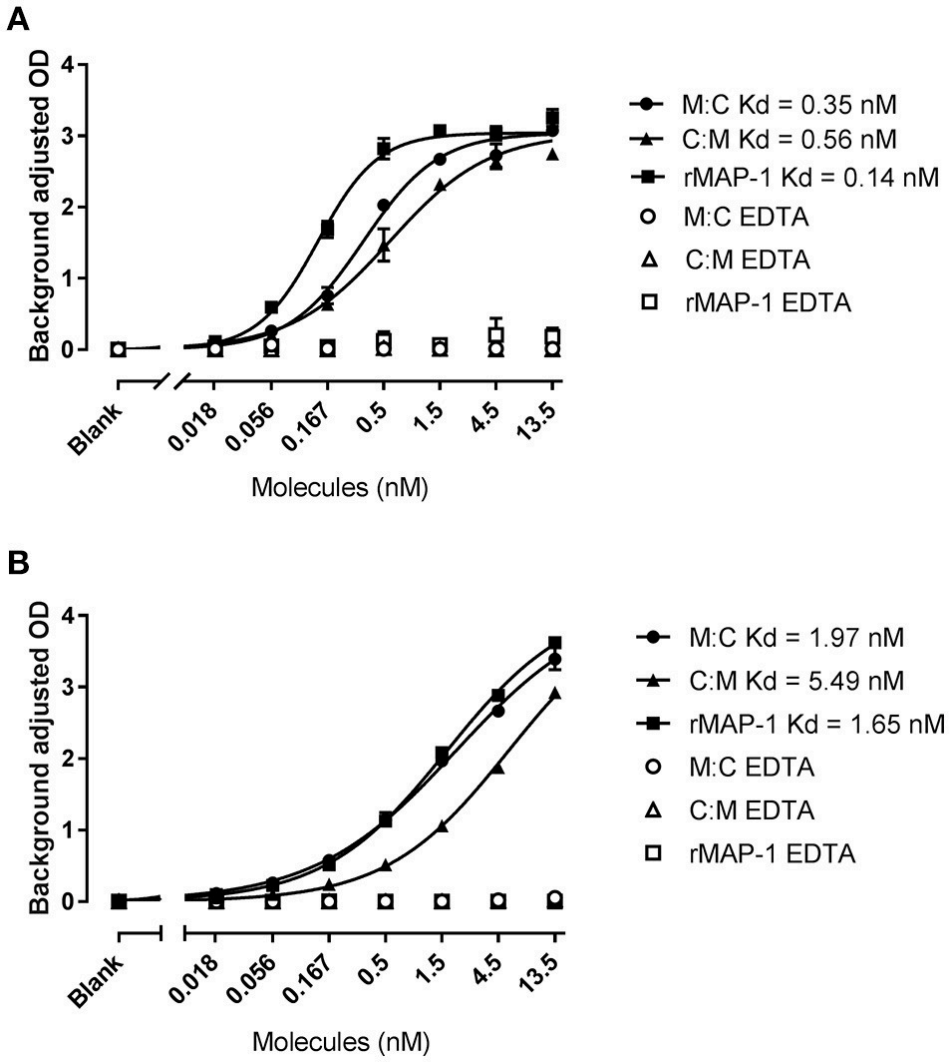
MAP-1:C4BP^1−5^ (M:C), C4BP^1−5^:MAP-1 (C:M) and MAP-1 binding to MBL **(A)** and CL-11 **(B)**. rMBL and rCL-11 were immobilized onto mannan-coated plates. Recombinant proteins were applied in a two-fold dilution in the presence of calcium or EDTA. Binding was determined using an anti-MAP-1 mAb. Connecting lines display nonlinear fitting using the equation specific binding with hill slope. Results are representative of three independent experiments and error bars represent minimum and maximum values of triplicate measurements.

Both chimeric proteins and MAP-1 were able to bind to rMBL and rCL-11 in a dose-dependent manner with equivalent dose-response curves in the presence of Ca^2+^ (Figure 3). On rMBL, MAP-1:C4BP^1−5^ and C4BP^1−5^:MAP-1 exhibited a similar Kd of 0.35 and 0.56 nM respectively, slightly lower affinity than rMAP-1 at the given conditions (0.14 nM; Figure 3A). The same tendency, albeit with weaker interactions, was observed with rCL-11 (Figure 3B): rMAP-1 displayed the lowest Kd (1.65 nM), followed by MAP-1:C4BP^1−5^ (1.97 nM) and lastly C4BP^1−5^:MAP-1 (5.49 nM). As expected, no binding could be detected when the proteins were incubated in the presence of EDTA.

### Inhibition of LP activation with MBL defect serum and NHS

**Figure 4:**
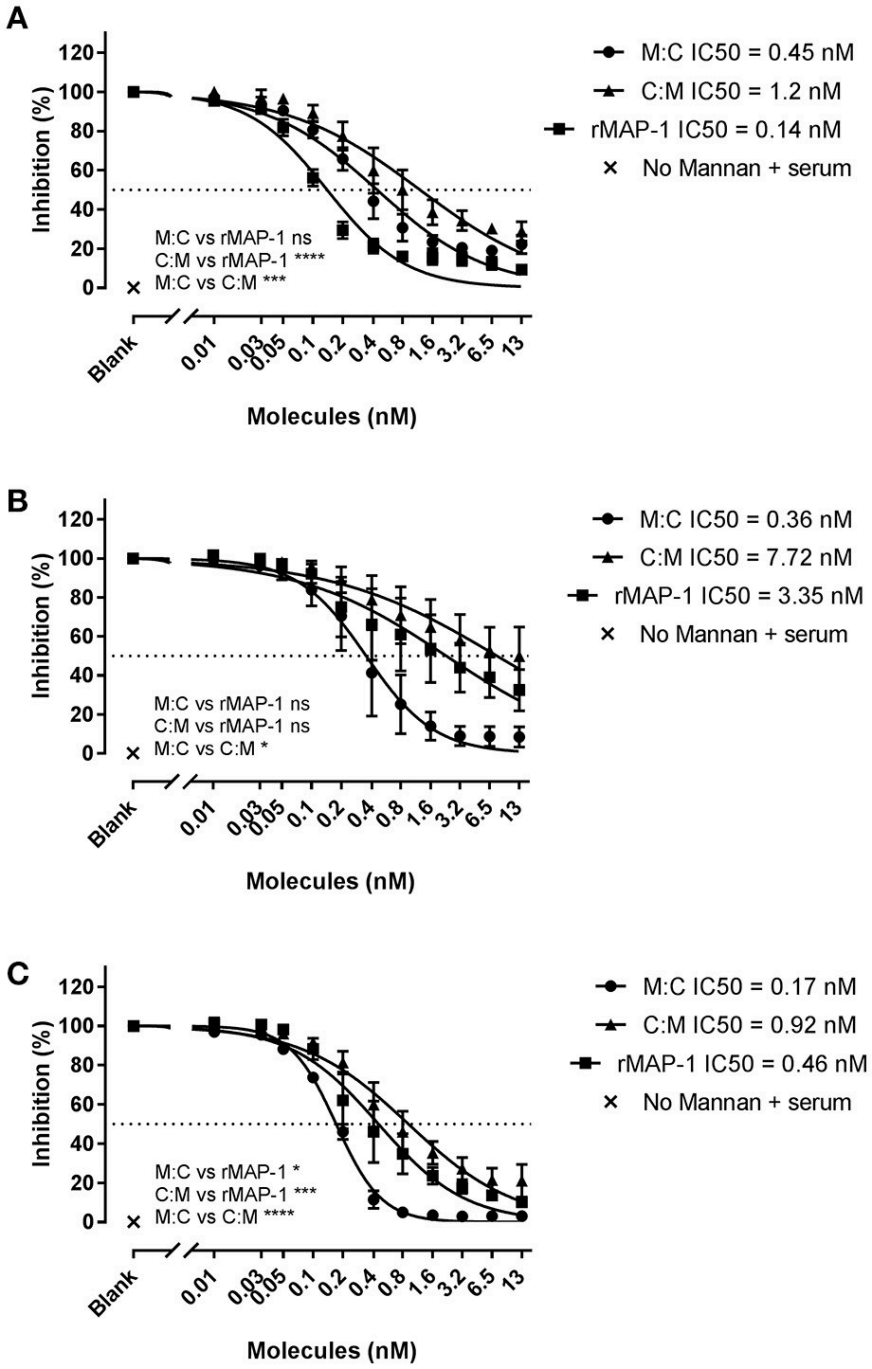
Chimeric and rMAP-1 complement inhibition after pre-incubation on rMBL/mannan. Serial dilutions of MAP-1:C4BP^1−5^ (M:C), C4BP^1−5^:MAP-1 (C:M), and rMAP-1 were allowed to form complexes with mannan-bound rMBL prior to addition of MBL-defect serum as a source of complement. Detection of C4 **(A)**, C3 **(B)**, and TCC **(C)** deposition was quantified using anti-C4, anti-C3, and anti-TCC mouse mAbs. Connecting lines are four parameters nonlinear fitting using the equation inhibitor concentration vs. slope. Data are reported as [(OD_inhibitor_-OD_background_)/(OD_noinhibitor_-OD_background_)]100. Error bars represent the SEM of three independent experiments, and the dashed line the 50% inhibition level. *P < 0.05; ***P < 0.001; ****P < 0.0001.

To study the effect of the chimeric constructs on LP-mediated complement activation we used mannan-bound rMBL as the activating PRM. MAP-1:C4BP^1−5^ and C4BP^1−5^:MAP-1 exhibited a strong dose-dependent inhibition on C4, C3, and TCC level when they were allowed to form complexes with rMBL prior to the addition of 2% MBL defect serum as complement source (Figure 4). rMAP-1 was the most effective at the C4 level: IC50 MAP-1 (0.14 nM) vs. IC50 MAP-1:C4BP^1−5^ (0.45 nM), non-significant; IC50 MAP-1 (0.14 nM) vs. IC50 C4BP^1−5^:MAP-1 (1.2 nM), *P* < 0.0001. Nonetheless, MAP-1:C4BP^1−5^ outperformed the rest when measuring C3 (IC50 MAP-1:C4BP^1−5^ = 0.36 nM vs. 7.72 nM C4BP^1−5^:MAP-1, *P* < 0.05, and 3.35 nM MAP-1, non-significant) and TCC deposition (IC50 MAP-1:C4BP^1−^5 = 0.17 nM vs. 0.92 nM C4BP^1−5^:MAP-1, *P* < 0.0001, and 0.46 nM MAP-1, *P* < 0.05).

**Figure 5:**
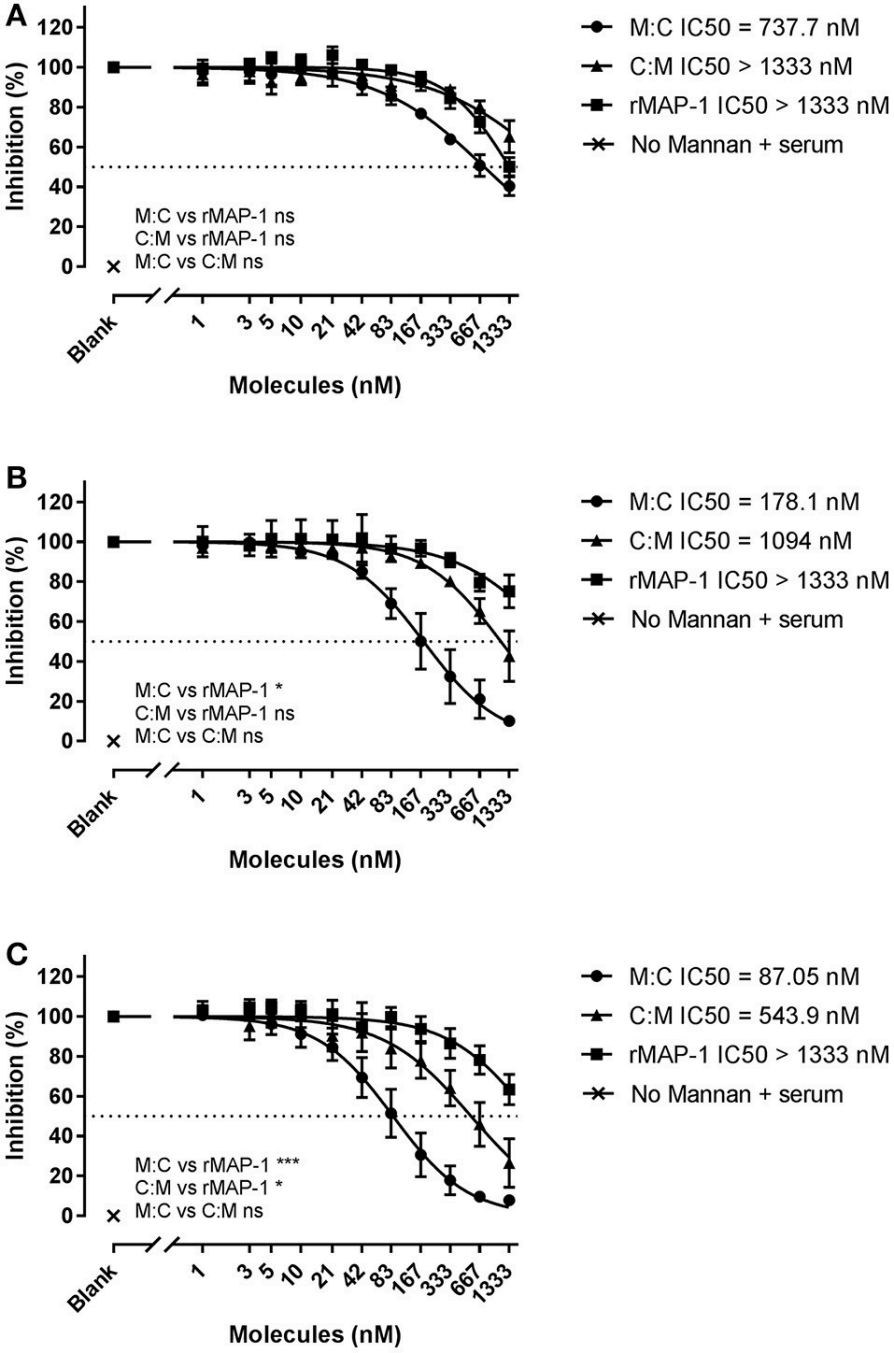
Chimeric and rMAP-1 complement inhibition after co-incubation with NHS. Serial dilutions of MAP-1:C4BP^1−5^ (M:C), C4BP^1−5^:MAP-1 (C:M), and rMAP-1 were co-incubated with 2% NHS in non-adsorbent titration plates for 30 min prior to addition to mannan-bound rMBL. Detection of deposition of C4 **(A)**, C3 **(B)**, and TCC **(C)** was determined as described before. Connecting lines are four parameters nonlinear fitting using the equation inhibitor concentration vs. slope. Dashed line Data are reported as [(OD_inhibitor_ – OD_background_)/(OD_noinhibitor_ – OD_background_)]100. Error bars represent the SEM of three independent experiments, and the dashed line the 50% inhibition level. *P < 0.05; ***P < 0.001.

Next, we tested the inhibitors by directly incubating them with 2% NHS in non-adsorbent titration plates before applying the inhibitor/serum mix to mannan (Figure 5). Under these conditions, both chimeric inhibitors demonstrated higher inhibition than rMAP-1 alone in the later steps of the complement cascade (i.e., C3 and TCC formation). MAP-1:C4BP^1−5^ exhibited a more pronounced inhibition of C4 (non-significant tendency), C3 (*P* < 0.05), and TCC deposition (*P* < 0.001). The other construct appeared to be less active but still outperformed rMAP-1 at the C3 (non-significant trend) and TCC levels (*P* < 0.05). No complement deposition was observed when MBL defect serum was incubated without rMBL (Figure 4) or with rMBL but no mannan (Figure 5).

### C4BP cofactor activity in FI-mediated cleavage of soluble C4b/C3b

It has previously been reported that C4BP can act as a cofactor in the fI-mediated proteolytic inactivation of C3b and C4b (29, 31, 49). Hence we examined whether our chimeric proteins retained the fI cofactor activity of native C4BP.

**Figure 6:**
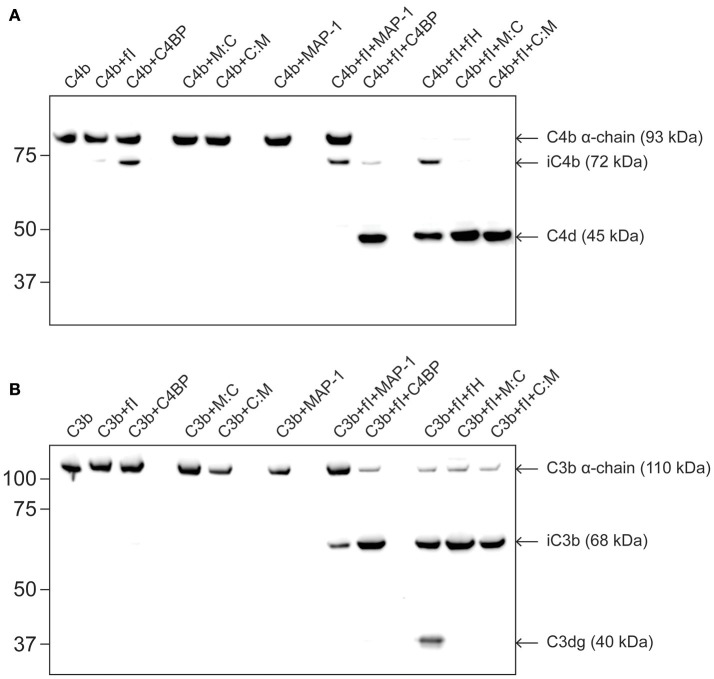
Cofactor activity in factor I-mediated C4b and C3b cleavage. Purified C3b or C4b was incubated with fI and different cofactors for 2 h at 37°C. The reactions were stopped with LDS buffer and subjected to western blotting under reducing conditions using C4d and C3d antibodies. **(A)** Incubation of C4b with fI in the presence of C4BP, fH or C4BP-containing chimeric proteins resulted in the generation of a band corresponding to the inactive degradation products iC4b and C4d. **(B)** Incubation of C3b with fI in the presence of C4BP, fH or C4BP-containing chimeric proteins results in the generation of the 68 kDa degradation fragment. M:C, MAP-1:C4BP^1−5^; C:M, C4BP^1−5^:MAP-1. The blots are representative of three independent experiments.

When C4b was incubated with fI in combination with native full length C4BP or one of the two chimeric proteins, the α-chain of C4b was cleaved and a band corresponding to the smaller cleavage product C4d appeared (Figure 6A). As expected no full cofactor activity was observed for MAP-1 but the cleavage product iC4b was observed when MAP-1 was in combination with FI and C4b. Similarly, a low level proteolysis of C3b into iC3b could be observed in the presence of fI and MAP-1. Both are probably artifacts from the elevated fI concentrations used in the assays. No C4b proteolysis was apparent in the absence of cofactors or fI, with the notable exception of purified native C4BP. When C4b was incubated with C4BP alone a cleavage of the α-chain and generation of the iC4b was observed, hinting at a possible contamination of the purified C4BP. We performed a new immunoblotting with C4BP alone and in combination with fI or C4b. The iC4b band was visible in all wells, suggesting that iC4b had been co-purified with C4BP (data not shown).

A cleavage of the C3b α-chain band was observed together with the appearance of a band corresponding to iC3b when C3b was subjected to fI in combination with C4BP or one of the two chimeric proteins (Figure 6B). Full cleavage of C3b to C3dg could only be archived when fH was applied in combination with C3b and fI. As expected no cofactor activity was observed for MAP-1.

### Reduction of MBL-dependent C4 deposition in kidney proximal tubular epithelial cells

**Figure 7:**
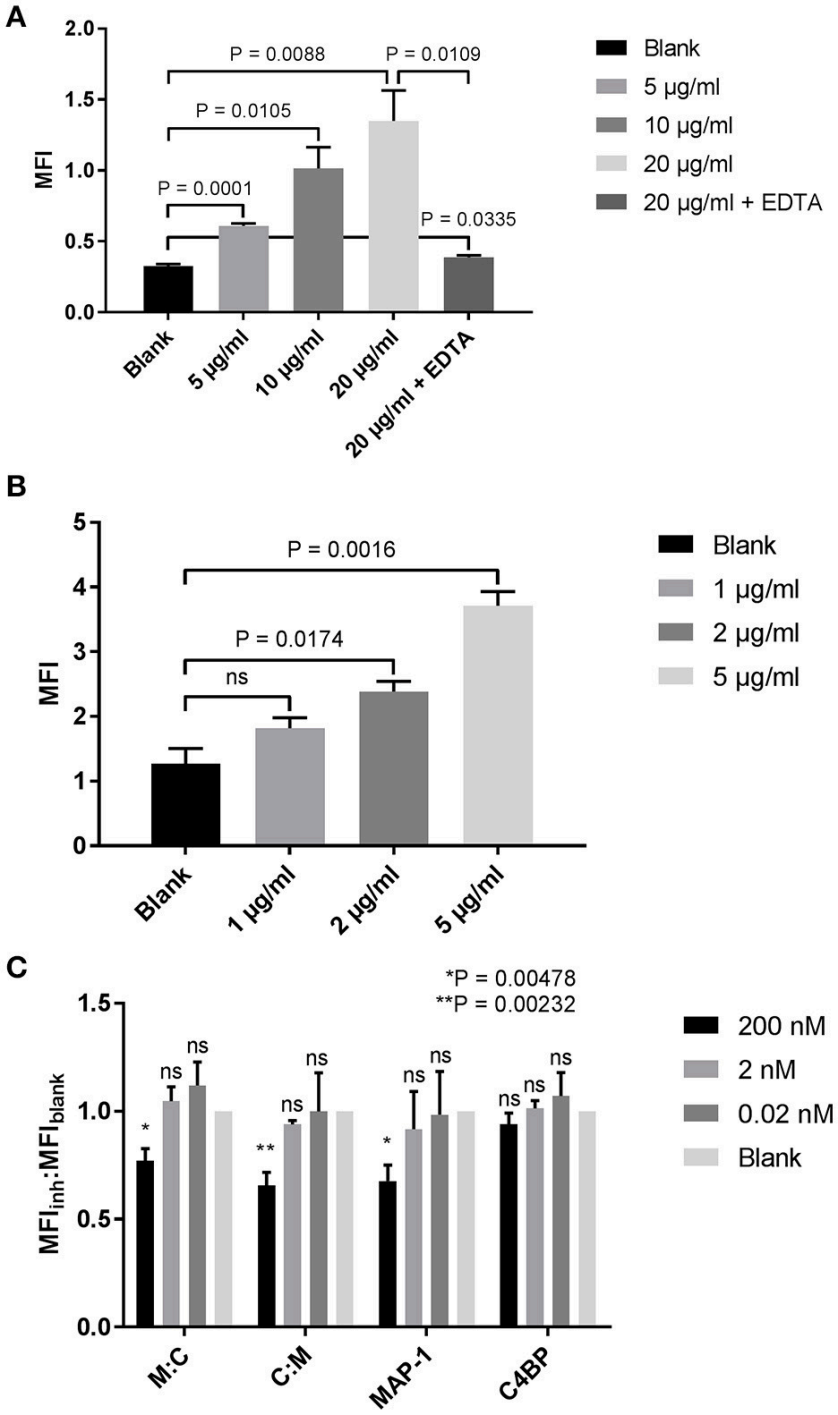
Inhibition of complement deposition on tubular proximal kidney epithelial cells. **(A)** HK-2 cells were incubated with increasing concentrations of rMBL at 4°C and bound MBL was determined using an anti-MBL mAb. **(B)** Binding of MBL results in the deposition of C4. HK-2 cells were incubated with rMBL followed by 10% MBL defect serum for 1 h at 4°C. Bound C4 was determined using an anti-C4c pAb. **(C)** rMBL-mediated C4 deposition after MAP-1:C4BP^1−5^ (M:C), C4BP^1−5^:MAP-1 (C:M), rMAP-1 or full length C4BP co-incubation with 10% MBL defect serum. Inhibition is reported as the ratio of the MFI of the inhibitor to the MFI of the bank (MFI_inh_:MFI_blank_). Significance was tested for each concentration of the inhibitors compared to the blank. Data are reported as the mean ± SEM of three independent experiments.

The complement system, and in particular MBL, has been documented as one of the key mediators of kidney injury following renal ischemia (50–53). Proximal tubular epithelial cells (PTECs), responsible for many regulatory and endocrine functions, are especially vulnerable to complement-mediated tissue damage (51). After demonstrating the efficacy of our chimeric inhibitors in the previous ELISA-based assays, we tested whether we could quench complement activation on PTECs. A dose-dependent MBL binding was observed when human PTECs were incubated with increasing concentrations of rMBL (Figure 7A). Binding could be neutralized with the addition of EDTA in the sample buffer, suggesting a classical c-type lectin interaction. Addition of 10% MBL defect serum to MBL-bound PTECs led to C4 deposition on the cell surface (Figure 7B) that was significantly reduced when rMAP-1 or the MAP-1-containing chimeras were incubated in a concentration of 200 nM with serum prior to addition to the MBL-bound cells as compared to the no inhibitor control (Figure 7C). Equimolar concentrations of rC4BP^1−5^ alone showed no significant reduction of C4 deposition.

### Total complement screen

**Figure 8:**
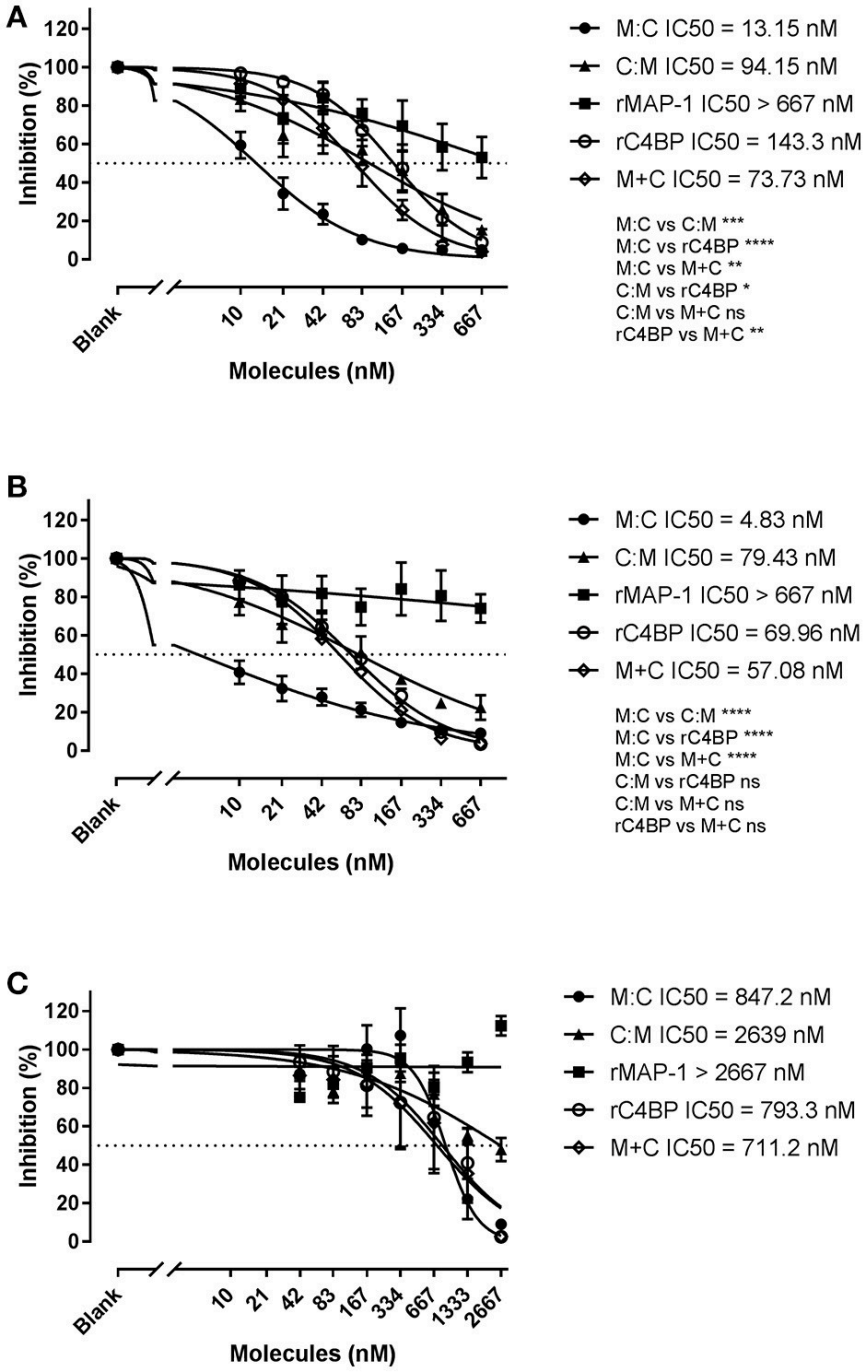
Wielisa total complement screen. Serial dilutions of MAP-1:C4BP^1−5^ (M:C), C4BP^1−5^:MAP-1 (C:M), rMAP-1, rC4BP^1−5^, and rMAP-1 and rC4BP^1−5^ together (M+C) were incubated with NHS and subsequently the protein/serum mix was applied to pre-coated Wielisa plates. Complement activation was quantified using an anti-TCC mAb. **(A)** Lectin pathway activation on mannan-coated plates. **(B)** Classical pathway activation on IgM-coated plates. **(C)** Alternative activation on LPS-coated plates. Connecting lines are nonlinear fitting using the equation inhibitor concentration vs. slope. Values represent OD readings normalized to the OD of the wells without inhibitor times 100. Significance was tested for best-fit IC50 values of data sets with an adjusted R-squared > 0.8. Error bars represent the SEM of three independent experiments, and the dashed line the 50% inhibition level. *P < 0.05; **P < 0.01; ***P < 0.001; ****P < 0.0001.

We further assessed the ability of the chimeric inhibitors to regulate all three complement pathways using the commercial total complement screen assay Wielisa (45) (Figure 8). In agreement with the above results MAP-1:C4BP^1−5^ was the most effective construct, significantly outperforming all other inhibitors in the LP (Figure 8A) and CP (Figure 8B) activation assays, while C4BP^1−5^:MAP-1 demonstrated a more modest activity. The combination of rC4BP^1−5^ and rMAP-1 displayed an inhibitory potential comparable to C4BP^1−5^:MAP-1, and 5 to 10 times lower than MAP-1:C4BP^1−5^. In the AP however (Figure 8C), MAP-1:C4BP^1−5^ had only a minor effect, with an IC50 value comparable to rC4BP^1−5^ (alone or in combination with rMAP-1). Inhibitors whose dose-response curves had a fitting with an adjusted R-square value < 0.8 were excluded for the analysis—such as rMAP-1 (Figures 8A,B) and all inhibitors besides MAP-1:C4BP^1−5^ in the AP activation assay (Figure 8C).

## Discussion

The unique position of the complement system as an early danger sensor and conductor of downstream humoral and cellular responses makes complement modulation an attractive pharmacological target (36, 54). Rational engineering of existing complement regulators and novel recombinant inhibitors targeting complement on different levels has proven to be a successful strategy, such as mini-fH (41) and chimeric regulators composed of the N-terminal domains of fH merged with CRIg (55) or soluble CR2 (40). Complement factor H is the main soluble regulator of the AP and with CRIg being a C3 regulator and CR2 an inhibitor of C3 deposition all these molecules affect exclusively the alternative amplification loop. However, increasing understanding of the involvement of complement in highly diverse clinical conditions suggests a different approach with complement-targeted drugs interfering at various stages of the cascade (35). Our group has previously tested this strategy by combining full length MAP-1 with the first five CCPs of fH, generating a chimeric protein with the potential to target initial recognition and at the same time down-regulate AP amplification (37). We wanted to investigate this approach further and designed two differently-oriented proteins by genetic engineering; MAP-1:C4BP^1−5^, with the sequence of human C4BP^1−5^ fused directly downstream of full length human MAP-1, and C4BP^1−5^:MAP-1, where C4BP^1−5^ was located in the N-terminus and connected to MAP-1 via a flexible glycine-serine linker. In theory, combining two existing complement inhibitors could result in a synergistic effect, giving rise to unique complement-regulatory and anti-inflammatory properties. Furthermore, we hypothesize that the ability of targeting both CP and LP activation may be especially beneficial in multifactorial pathologies where both pathways are involved in driving tissue damage, such as ischemia/reperfusion injuries (56).

MAP-1 forms head-to-tail homodimers (18) stabilized by calcium, that upon complex formation with collectins and ficolins downregulate LP activation (18, 57, 58). MAP-1:C4BP^1−5^, C4BP^1−5^:MAP-1, and rMAP-1 displayed comparable Kd values to rMBL (0.35, 0.56, 0.14 nM respectively) and rCL-11 (1.97, 5.49, and 1.65 nM respectively) in a solid-phase ELISA setup. Hill slopes values above 1 indicate a multivalent binding to MBL. The lower hill slope values observed with rCL-11 may be a consequence of its limited oligomerization and reflect the reduced number of collagen-stalks available for complexation with the inhibitors. Addition of the calcium-chelating agent EDTA completely inhibited binding. This clearly indicates that the interaction of the chimeras with the PRMs is mediated through their MAP-1 part in a calcium-dependent manner. No binding was observed with rC4BP^1−5^ (data not shown).

To investigate the dimerization in detail both chimeras were subjected to SEC in the presence of physiological calcium concentrations or EDTA. We found that both constructs eluted as a single peak in the presence of calcium, whereas when exposed to EDTA-containing buffers the chimeras were separated into monomers leading to a shift in their spectrophotogram profiles toward a lower estimated molecular size. The identity of the peaks was confirmed using a specific ELISA capturing C4BP and detecting MAP-1. These results demonstrate that the constructs maintain the calcium-dependent dimerization of native MAP-1 that is critical for its role as an LP inhibitor.

Our group and others have previously demonstrated that rMAP-1 alone and as part of chimeric constructs inhibits LP activation *in vitro* (18, 20, 21, 37, 39). Here we tested the ability of the chimeric proteins to regulate LP activation in different functional ELISAs. First we incubated the chimeric proteins, rMAP-1, and C4BP^1−5^ with MBL bound to mannan-coated plates, and subsequently added 2% MBL defect serum as complement source. While rMAP-1 alone seemed more effective at inhibiting C4 deposition, MAP-1: C4BP^1−5^ clearly outperformed rMAP-1 on the C3 and TCC levels. C4BP^1−5^:MAP-1 was the least effective regulator of the three. If C4 regulation is solely dependent on MAP-1, then differences on C4 inhibition could be explained by their different binding affinities toward MBL. In a more physiologically-relevant ELISA setup the proteins were co-incubated with 2% NHS, thus allowing direct competition between the inhibitors and intrinsic MASPs for binding to MBL. Again, incubation with MAP-1:C4BP^1−5^ caused a significant inhibition resulting in the almost complete absence of C3, and TCC deposition. While C4BP^1−5^:MAP-1 failed to provide the same inhibition as MAP-1:C4BP^1−5^, it was still more effective than rMAP-1 alone. The difference in inhibition levels of rMAP-1 observed in the two experimental setups is in agreement with other studies (18, 37) and could be attributed to competition with the MASPs on MBL binding. This phenomenon supports our explanation that rMAP-1 occupies MASPs-binding sites at the MBL molecules when pre-incubated (20), that would result in either a lower concentration of catalytically-active MASPs or/and a decreased intercomplex activation by spacing out MBL/MASPs complexes (59). When added simultaneously with serum, rMAP-1 may not be capable of displacing already formed MBL/MASPs complexes (at least under our experimental conditions).

It has been reported that the domains CCP1-4 of the α-chain are critical for the fI cofactor activity of C4BP in the fI-mediated C4b (28–30) and C3b proteolytic inactivation (31). Thereby we expected that the chimeric proteins will manifest intact fI cofactor activity. We showed that C4BP^1−5^-containing constructs were as efficient as full length native C4BP in a fluid phase C3b/C4b degradation assay. Moreover it appears that C4BP is a more effective cofactor in the cleavage of C4b compared to C3b in agreement with a previous study (49).

We next looked for a pathological condition where complement injury was triggered by an initial recognition by the LP. MBL has been documented to be directly involved in kidney damage following renal ischemia (53). We used immortalized human PTECs to test whether we could quench the exacerbated complement activation observed on the tubular epithelium, known to be especially vulnerable to complement attack after reperfusion (51). In agreement with the literature rMBL was capable of binding to the cell surface in a calcium-dependent manner, suggesting a traditional c-type lectin interaction (60). Binding to PTECs led to the deposition of C4 activation fragments. As seen on ELISA, the chimeras and rMAP-1 presented comparable regulatory effects (even when using a five times lower serum dilution than in ELISA). This to a degree limited inhibition can be expected assuming that trace amounts of PRM/MASPs complexes are sufficient to cause detectable C4 deposition, which in turn is mainly mediated by the MAP-1 part while the effect on the late cascade (i.e., C3 and TCC) depends on the combination of MAP-1 and C4BP. No C3 or TCC deposition was observed (see Supplementary Figure 2). The assay was performed at 4°C, that while it allowed the cleavage and deposition of C4, it did not result in the assembly of the downstream convertases [62]. Incubation of MBL-bound cells at 37°C led to the disappearance of C4 and MBL surface stain (data not shown). It has been reported that binding of MBL to PTEC causes its internalization followed by complement-independent cell death (60). Thus, this assay may have some inherited limitations to study downstream complement deposition, but is suitable to study the initial inhibiting effect of PRM binding inhibitors.

Finally, we tested the regulatory effect of the chimeric proteins in all three complement pathways using Wielisa, a platform for standardized complement activity measurements. In agreement with our previous results, MAP-1:C4BP^1−5^ demonstrated a strong dose-dependent inhibition of TCC deposition in the LP (IC50 = 13.15 nM). Remarkably, it was also an efficient CP inhibitor (IC50 = 4.83 nM), outperforming rMAP-1 and C4BP^1−5^ alone or in combination. C4BP^1−5^:MAP-1 inhibited the LP and CP to a lesser extent than MAP-1:C4${\text{BP}}_{,}^{1-5}$ equivalent to rMAP-1 and rC4BP^1−5^ added at the same time. The equivalent regulatory effects of the chimeras in the LP and CP, together with the moderate activity of rMAP-1 in the LP, suggests that the observed LP-downregulation is mainly driven by the C4BP part, reflecting a limitation of the assay as a model for LP-mediated activation. Thus, in a physiological setting where both functional domains of the chimeras would be involved, we may observe a more pronounced inhibition [as shown for rMAP-1 (21)]. It should be noted that even though MAP-1 only associates with the PRMs of the LP and not the CP, MAP-1-containing chimeras were more effective than C4BP^1−5^ alone in both CP and LP. This implies that the combination of two regulators within a single molecule—in the right configuration—enables the enhanced functionality of the constructs, and not just displays the function of “free” MAP-1 or C4BP. In the AP activation assay MAP-1:C4BP^1−5^ showed only a minor effect—around 100 to 400-fold lower than in the LP and CP—that was directly comparable to rC4BP^1−5^ (alone or in combination with rMAP-1), suggesting that AP activity is solely mediated by the C4BP part. It is important to highlight that the above-described experiments were performed using diluted serum as a source of complement. Experiments using animal models would be necessary to demonstrate that the chimeric proteins are effective complement inhibitors under physiological conditions.

The mechanism behind the observed differences in inhibitory properties between MAP-1:C4BP^1−5^ and C4BP^1−5^:MAP-1 remain unclear, although it could be explained by their quaternary structure. Dimerization of C4BP^1−5^:MAP-1 could result in MAP-1 bending away from C4BP^1−5^ via the flexible region in the CUB2/EGF region and the artificial linker between C4BP^1−5^ and MAP-1. This contorted folding could give rise to a dimer with impaired ability to reach C4b/C3b and possibly also hindering the complex formation with MBL. On the other hand, MAP-1:C4BP^1−5^ may fold into an elongated dimer with C4BP^1−5^ pointing outwards from a MAP-1 dimer core bound to the collagen-like stalks of the PRMs similar to how the serine domain in the MASPs is assumed to protrude when bound to MBL. This conformation would enable both C4BP^1−5^ monomers to interact with deposited C4b/C3b in the vicinity of the PRM binding site. We propose that the MAP-1 fragment may “dock” the chimeric inhibitor to the PRMs directing the multifunctional soluble inhibitor C4BP to the activating surface, thus increasing the efficacy. While data presented by our group in the present and past reports (37) provide a solid support for our hypothesis, conclusive evidence will require precise structural determination techniques, such as circular dichroism, multi-angle light scattering, small-angle x-ray scattering, or x-ray crystallography.

In conclusion, we have successfully developed a novel complement inhibitor that harnesses the multifaceted functionality of the fluid phase CP and LP regulator C4BP, and directs it to danger foci by the specific association to PRMs mediated by MAP-1. The combination of MAP-1 fused to C4BP^1−5^ presents unique complement regulatory properties *in vitro* and represents a potential novel therapeutic approach by targeting both upstream and central complement activation.

## Author contributions

CH, RB-O, NK-M, M-OS, and SvP performed the experiments. KP optimized the ELISA-based inhibition assays. RB-O, M-OS, and PG designed the study. CH, RB-O, PG wrote the manuscript. All authors critically reviewed the manuscript.

### Conflict of interest statement

The authors declare that the research was conducted in the absence of any commercial or financial relationships that could be construed as a potential conflict of interest.

## Supplementary material

Supplementary Figure 1 Gating strategy. HK-2 cells were defined as a uniform population on the forward scatter (FS INT) vs. side scatter (SS INT) plot, and single cells were selected gating on the forward scatter area or integral values (FS INT) vs. forward scatter height or peak (FS PEAK).

Supplementary Figure 2 MBL-dependent complement deposition on HK-2 cells. Cells were incubated with rMBL for 30 min at 4°C prior to addition of 10% MBL defect serum for 1 h at 4°C. Deposition of C4 **(A)**, C3 **(B)**, and TCC **(C)**. Cells incubated with no MBL, or with MBL but no serum were used as controls. X-Med, median fluorescence intensity.

## Abbreviations

CP, LP, AP: Classical, lectin and alternative pathway
PRM: Pattern recognition molecule
IC50: Half maximal inhibitory concentration
Kd: half maximal binding concentration
SEM: Standard error of the mean
PTEC: Proximal tubular epithelial cells.
