# Supplementary figures and images for: Chimeric Proteins Containing MAP-1 and Functional Domains of C4b-Binding Protein Reveal Strong Complement Inhibitory Capacities
Source: Front Immunol. 2018 Aug 28;9:1945. doi: 10.3389/fimmu.2018.01945 (PMC6120983; doi:10.3389/fimmu.2018.01945)

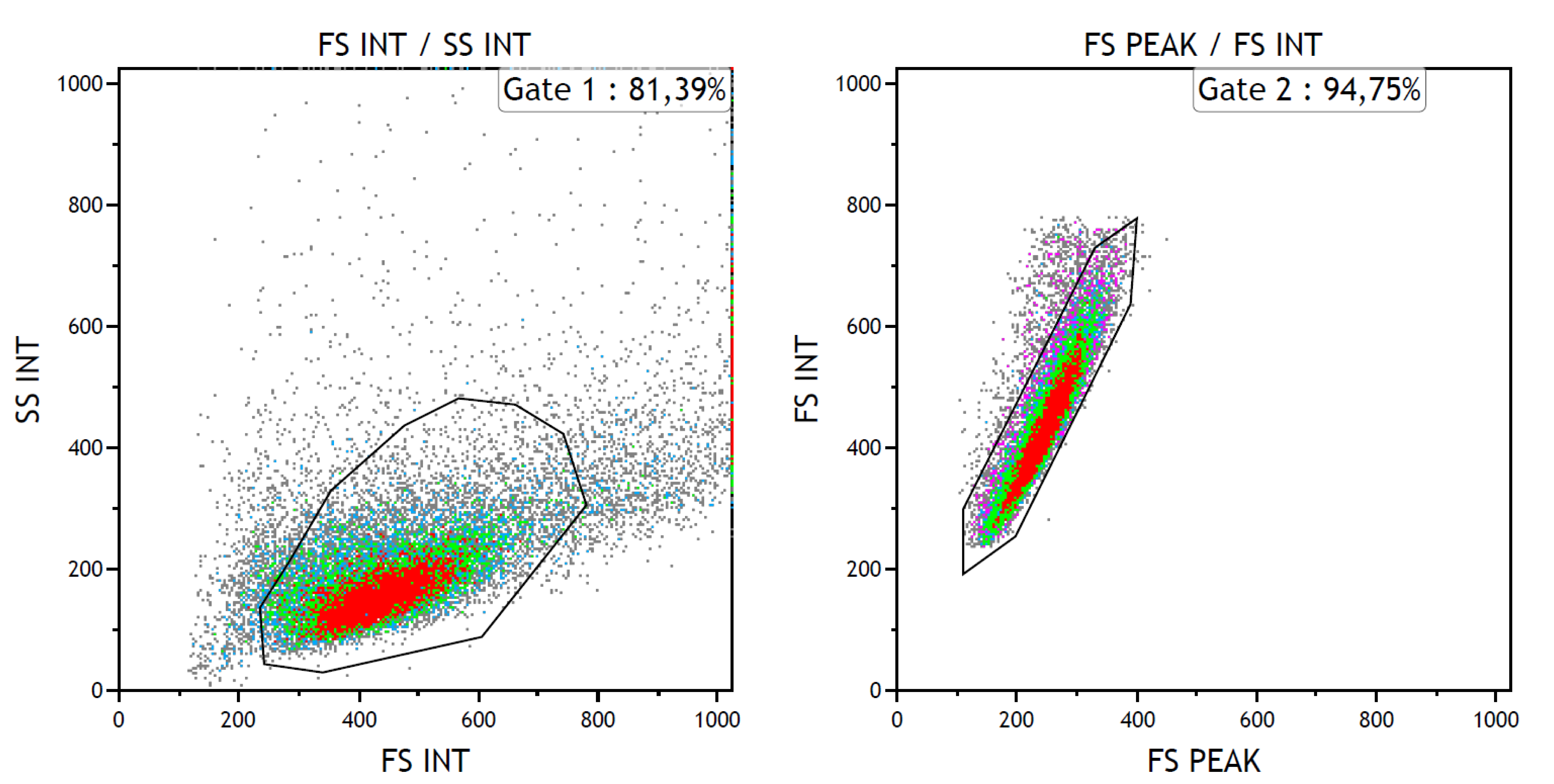

Supplement: Supplementary Figure 1 — Gating strategy. HK-2 cells were defined as a uniform population on the forward scatter (FS INT) vs. side scatter (SS INT) plot, and single cells were selected gating on the forward scatter area or integral values (FS INT) vs. forward scatter height or peak (FS PEAK). [file Image_1.TIF]

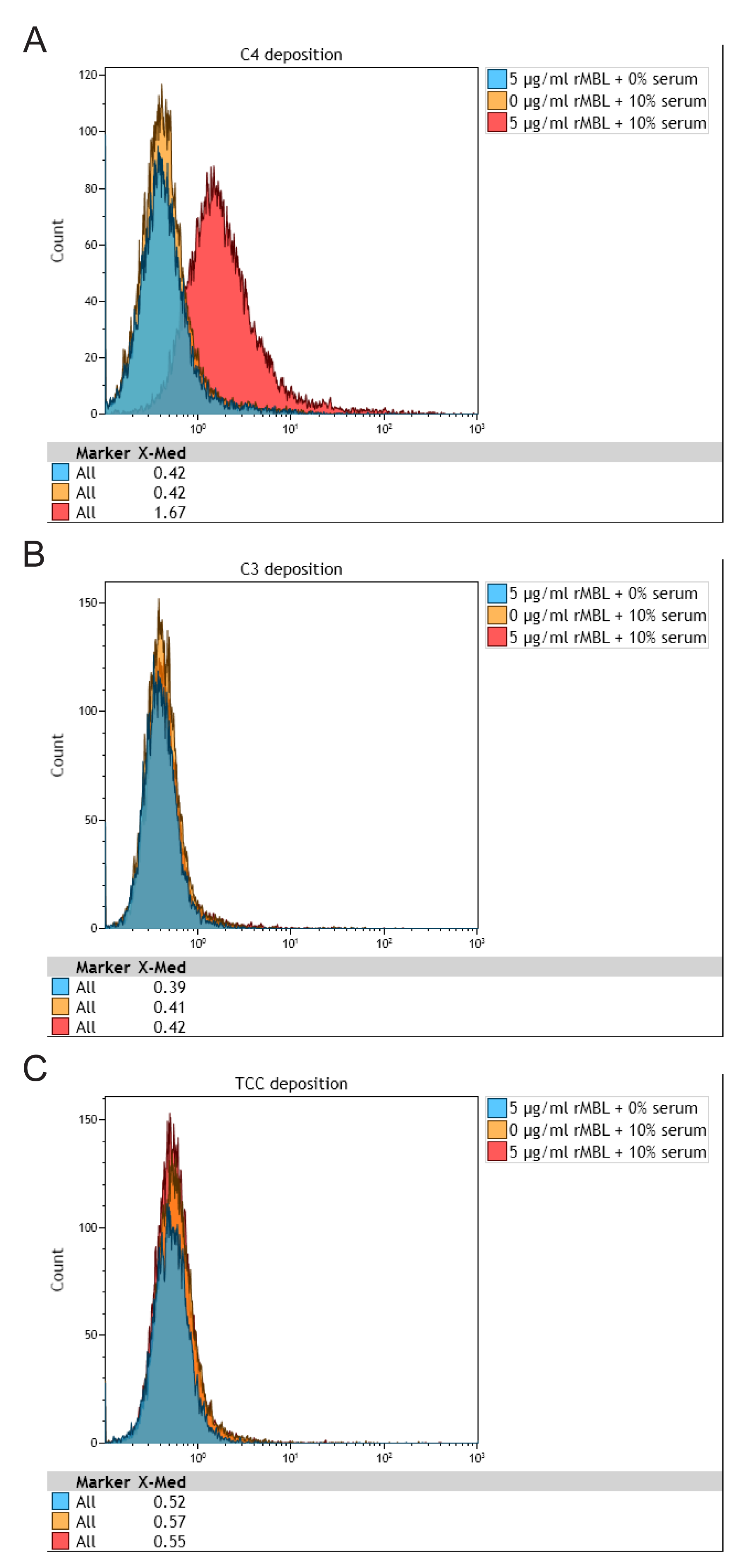

Supplement: Supplementary Figure 2 — MBL-dependent complement deposition on HK-2 cells. Cells were incubated with rMBL for 30 min at 4°C prior to addition of 10% MBL defect serum for 1 h at 4°C. Deposition of C4 (A), C3 (B), and TCC (C). Cells incubated with no MBL, or with MBL but no serum were used as controls. X-Med, median fluorescence intensity. [file Image_2.TIF]
